# Lactoferricin Peptides Increase Macrophages' Capacity To Kill *Mycobacterium avium*

**DOI:** 10.1128/mSphere.00301-17

**Published:** 2017-08-30

**Authors:** Tânia Silva, Ana C. Moreira, Kamran Nazmi, Tânia Moniz, Nuno Vale, Maria Rangel, Paula Gomes, Jan G. M. Bolscher, Pedro N. Rodrigues, Margarida Bastos, Maria Salomé Gomes

**Affiliations:** a i3S, Instituto de Investigação e Inovação em Saúde, Universidade do Porto, Porto, Portugal; b Instituto de Biologia Molecular e Celular (IBMC), Universidade do Porto, Porto, Portugal; c Centro de Investigação em Química, Departamento de Química e Bioquímica, Faculdade de Ciências, Universidade do Porto, Porto, Portugal; d Instituto de Ciências Biomédicas Abel Salazar (ICBAS), Universidade do Porto, Porto, Portugal; e Department of Oral Biochemistry, Academic Centre for Dentistry Amsterdam (ACTA), University of Amsterdam, and VU University Amsterdam, Amsterdam, The Netherlands; f REQUIMTE-UCIBIO, Departamento de Química e Bioquímica, Faculdade de Ciências, Universidade do Porto, Porto, Portugal; University of Rochester

**Keywords:** autophagy, lactoferricin, *Mycobacterium*, antimicrobial peptide, macrophage

## Abstract

Mycobacterial infections cause a significant burden of disease and death worldwide. Their treatment is long, toxic, costly, and increasingly prone to failure due to bacterial resistance to currently available antibiotics. New therapeutic options are thus clearly needed. Antimicrobial peptides represent an important source of new antimicrobial molecules, both for their direct activity and for their immunomodulatory potential. We have previously reported that a short version of the bovine antimicrobial peptide lactoferricin with amino acids 17 to 30 (LFcin17–30), along with its variants obtained by specific amino acid substitutions, killed *Mycobacterium avium* in broth culture. In the present work, those peptides were tested against *M. avium* living inside its natural host cell, the macrophage. We found that the peptides increased the antimicrobial action of the conventional antibiotic ethambutol inside macrophages. Moreover, the d-enantiomer of the lactoferricin peptide (d-LFcin17–30) was more stable and induced significant killing of intracellular mycobacteria by itself. Interestingly, d-LFcin17–30 did not localize to *M. avium*-harboring phagosomes but induced the production of proinflammatory cytokines and increased the formation of lysosomes and autophagosome-like vesicles. These results lead us to conclude that d-LFcin17–30 primes macrophages for intracellular microbial digestion through phagosomal maturation and/or autophagy, culminating in mycobacterial killing.

**IMPORTANCE** The genus *Mycobacterium* comprises several pathogenic species, including *M. tuberculosis*, *M. leprae*, *M. avium*, etc. Infections caused by these bacteria are particularly difficult to treat due to their intrinsic impermeability, low growth rate, and intracellular localization. Antimicrobial peptides are increasingly acknowledged as potential treatment tools, as they have a high spectrum of activity, low tendency to induce bacterial resistance, and immunomodulatory properties. In this study, we show that peptides derived from bovine lactoferricin (LFcin) improve the antimicrobial activity of ethambutol against *Mycobacterium avium* growing inside macrophages. Moreover, the d-enantiomer of a short version of lactoferricin containing amino acids 17 to 30 (d-LFcin17–30) causes intramacrophagic death of *M. avium* by increasing the formation of lysosomes and autophagosomes. This work opens the way to the use of lactoferricin-derived peptides to treat infections caused by mycobacteria and highlights important modulatory effects of d-FLcin17–30 on macrophages, which may be useful under other conditions in which macrophage activation is needed.

## Abstract (precis)

The genus *Mycobacterium* comprises several pathogenic species, including *M. tuberculosis*, *M. leprae*, *M. avium*, etc. Infections caused by these bacteria are particularly difficult to treat due to their intrinsic impermeability, low growth rate, and intracellular localization. Antimicrobial peptides are increasingly acknowledged as potential treatment tools, as they have a high spectrum of activity, low tendency to induce bacterial resistance, and immunomodulatory properties. In this study, we show that peptides derived from bovine lactoferricin (LFcin) improve the antimicrobial activity of ethambutol against *Mycobacterium avium* growing inside macrophages. Moreover, the d-enantiomer of a short version of lactoferricin containing amino acids 17 to 30 (d-LFcin17–30) causes intramacrophagic death of *M. avium* by increasing the formation of lysosomes and autophagosomes. This work opens the way to the use of lactoferricin-derived peptides to treat infections caused by mycobacteria and highlights important modulatory effects of d-FLcin17–30 on macrophages, which may be useful under other conditions in which macrophage activation is needed.

## INTRODUCTION

The *Mycobacterium* genus contains several species capable of causing severe disease, such as those belonging to the *M. tuberculosis* complex, *M. leprae*, and nontuberculous mycobacteria (NTM) (1, 2). The incidence of NTM infections, predominantly by species of the *M. avium* complex (MAC), is increasing worldwide, surpassing in some regions the number of infections caused by *M. tuberculosis* (3, 4). Disseminated infections caused by NTM occur mainly in patients with a compromised immune system, such as HIV-infected patients, patients with cancer, and organ or stem cell transplant patients, among others (reviewed in references 2, 5, and 6).

Mycobacteria are characterized by a unique, complex, highly impermeable cell wall and are able to proliferate inside phagocytic cells, subverting the intracellular vesicular trafficking. These characteristics confer upon them high resistance to chemotherapy and the ability to cause persistent infections (7, 8). Treatment regimens are based on a combination of several drugs taken for months to years and in general have limited efficacies (9, 10). Furthermore, mycobacterial antibiotic resistance is increasing worldwide, urging the need to develop novel classes of antimicrobial drugs (11).

Mycobacteria are facultative intracellular pathogens residing mainly inside macrophages. After being phagocytized, the mycobacteria arrest the maturation of the phagosome, inhibiting the phagosome-lysosome fusion (12–14). This inhibition enables mycobacteria not only to escape the harmful environment of lysosomes but also to maintain the interaction with endosomes in the recycling pathway, allowing their access to nutrients [e.g., transferrin-bound Fe(III)] needed to ensure survival and proliferation inside the host (12, 15, 16). Cytokines such as gamma interferon (IFN-γ) and tumor necrosis factor alpha (TNF-α) play an important role in macrophage activation and mycobacterial growth restriction (17–20). However, the mechanisms by which macrophages inhibit mycobacterial growth and the mechanisms used by mycobacteria to resist and live inside macrophages are not fully understood. Respiratory burst and nitric oxide (NO) are involved in *M. tuberculosis* killing (21–23), but they do not seem to play an important role in the case of *M. avium* (15, 24, 25). Nutrient restriction, including that of iron, is also thought to have a role, namely, through alterations in vesicular trafficking that affect mycobacterium-harboring phagosomes (15). Cell death mechanisms are also important for cell homeostasis and infection control. In fact, mycobacteria are known to modulate pathways such as apoptosis, autophagy, necrosis, and pyroptosis, which have been implicated in infection containment but also in enhanced bacterial spread (26–28).

Antimicrobial peptides (AMP) are an important component of the innate immune response against pathogens. These peptides are widespread in nature as part of host defense mechanisms, constituting potential new antimicrobial treatment options (29). Although their mode of action is still under debate, they are thought to act with a multiple-hit strategy, which probably contributes to their high efficacy and large spectrum of activity. AMP can act directly on pathogens, either by disrupting the membrane due to pore formation and/or micellization or by acting on internal targets (30). They can also act by immunomodulation, being involved in several processes, such as modulation of pro- and anti-inflammatory responses, chemoattraction, cellular differentiation, angiogenesis, wound healing, enhancement of bacterial clearance, autophagy, and apoptosis, among others (31). In the case of mycobacteria, one of the most effective mechanisms of host resistance is the vitamin D-dependent induction of an AMP (LL-37) and autophagy (32–34).

Lactoferricin is an antimicrobial peptide obtained by pepsin digestion of the highly cationic N1 terminal domain of the iron-binding protein lactoferrin (35, 36). The bovine lactoferricin is composed of 25 amino acids (positions 17 to 41 in the native protein) (37) and has a broad-spectrum antimicrobial activity (reviewed in reference 38). A shorter version, with amino acids 17 to 30 (LFcin17–30), was found to have high antimicrobial activity against both Gram-positive and Gram-negative bacteria (39). We have previously shown that arginine residues are crucial for the antimicrobial activity of LFcin17–30 against *M. avium* growing in broth culture and that the d-enantiomer (d-LFcin17–30) was even more active than the l-enantiomer (40). In the present work, LFcin17–30 and its variants were tested against *M. avium* growing inside mouse macrophages, alone or in combination with the conventional antibiotic ethambutol. We found that the d-LFcin17–30 enantiomer was the most active peptide, acting through modulation of macrophages' defense mechanisms.

## RESULTS

### Up to 40 µM, lactoferricin peptides are not toxic to primary mouse macrophages.

**FIG 1:**
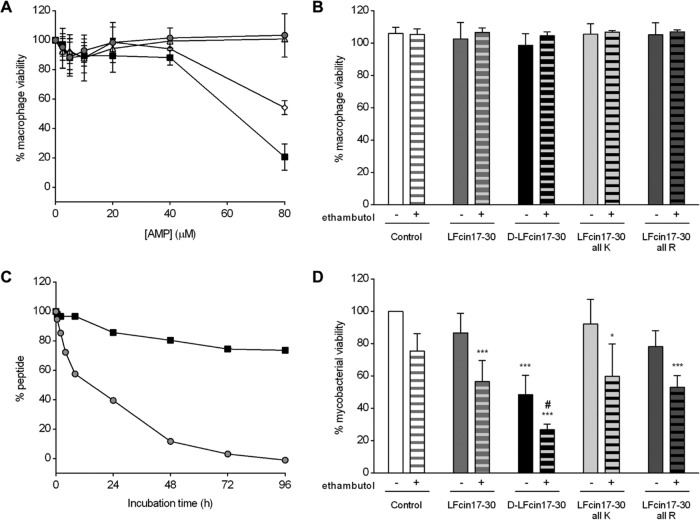
Effect of lactoferricin peptides on *M. avium*-infected macrophages. (A) BALB/c mouse BMM were infected with *M. avium* 2447 SmT and incubated with LFcin17–30 (gray circles), d‑LFcin17–30 (black squares), LFcin17–30 all K (white triangles), and LFcin17–30 all R (white diamonds) for 24 h. At the end of this period, 10% resazurin (125 µM) was added, and 24 h later fluorescence was measured at 560/590 nm to evaluate cell viability. The graph shows the averages ± standard deviations of results of two independent experiments, presented as percentages of viable cells relative to the number of corresponding non-peptide-treated infected cells. (B) BALB/c BMM were infected with *M. avium* 2447 SmT and treated with 40 µM LFcin17–30, d-LFcin17–30, LFcin17–30 all K, or LFcin17–30 all R alone (nonpatterned bars) or in combination with 7.2 µM ethambutol (patterned bars). After 5 days of incubation, 10% resazurin (125 µM) was added, and 24 h later fluorescence was measured at 560/590 nm to evaluate cell viability. The graph shows the averages + standard deviations of results from three independent experiments, presented as percentages of viable cells relative to the number of corresponding non-peptide-treated infected cells. (C) LFcin17–30 (gray circles) and d-LFcin17–30 (black squares) at a 40 µM final concentration were incubated with cell medium at 37°C. After 0, 0.5, 2, 4, 8, 24, 48, 72, and 96 h of incubation, a 40-µl aliquot was immediately injected for RP-HPLC analysis using an elution gradient of 0 to 100% acetonitrile in 0.05% aqueous trifluoroacetic acid (TFA) for 30 min at a flow rate of 1 ml/min. The results are presented as percentages of the remaining peptide in relation to the amount of peptide present at time zero. (D) BALB/c BMM were infected with *M. avium* 2447 SmT and treated with 40 µM LFcin17–30, d-LFcin17–30, LFcin17–30 all K, or LFcin17–30 all R alone (nonpatterned bars) or in combination with 7.2 µM ethambutol (patterned bars). After 5 days of incubation, bacteria were quantified by a CFU assay. The results represent the averages + standard deviations from at least four independent experiments and are expressed as the percentage of intramacrophagic mycobacteria in each well relative to the number of mycobacteria in the nontreated infected cells (control) in each experiment. Statistics were performed using two-way ANOVA with Tukey’s multiple-comparison test. *, *P* < 0.05; **, *P* < 0.01; ***, *P* < 0.001 compared to nontreated wells (control); #, *P* < 0.001 compared to ethambutol alone.

Previously (40), we showed that bovine LFcin17–30 and its variants with all arginines replaced with lysines and vice versa (LFcin17–30 all K and LFcin17–30 all R, respectively), as well as the variant with all amino acids in the d-form (d-LFcin17–30) (Table 1), killed *M. avium* in axenic cultures. In this work, we decided to investigate whether those peptides were able to kill mycobacteria growing inside macrophages, their natural host cells. We have also tested the possible synergistic effect of lactoferricin peptides with ethambutol, a conventional antibiotic used in the clinics to treat mycobacterial infections (41, 42). Before testing the compounds for their antimicrobial activity, we evaluated their potential toxicity toward bone marrow-derived macrophages (BMM). In Fig. 1A and B, we show that the peptides, alone or in combination with ethambutol, did not exert a significant toxic effect on noninfected (data not shown) or infected macrophages at 40 µM, 1 and 5 days after incubation.

**TABLE 1:** Characteristics of synthetic lactoferricin peptides

Peptide	Amino acid sequence	Molecular wt	Charge^a^
LFcin17–30	FKCRRWQWRMKKLG	1,923	+6
d-LFcin17–30	FKCRRWQWRMKKLG	1,923	+6
LFcin17–30 all K	FKCKKWQWKMKKLG	1,839	+6
LFcin17–30 all R	FRCRRWQWRMRRLG	2,007	+6

a Calculated overall charge at a pH of 7.0.

### Lactoferricin peptides inhibit *M. avium* growth inside macrophages and synergize with ethambutol.

Given that the peptides at up to 40 µM were not toxic to macrophages, we evaluated their effect on *M. avium* growing inside these cells. Bone marrow-derived macrophages were obtained from BALB/c mice and infected with *M. avium* 2447 smooth transparent variant (SmT). The different peptides were added at 40 µM, and ethambutol was added at 7.2 µM. After 5 days in culture, the number of intracellular bacteria per culture well was quantified in a CFU assay (Fig. 1D). Among the peptides tested, only d-LFcin17–30 significantly inhibited the intramacrophagic growth of *M. avium* (52% growth reduction, *P* < 0.001). None of the other peptides or ethambutol alone significantly inhibited *M. avium* growth. Interestingly, when given to the macrophages in combination with ethambutol, all peptides had a significant inhibitory effect, revealing a possible synergistic effect between antibiotic and AMP. Of note, even in combination with ethambutol, d-LFcin17–30 was still the most active peptide (73% reduction in *M. avium* growth relative to the control; *P* < 0.001).

### d-LFcin17–30 is more resistant to degradation by medium components than LFcin17–30.

To understand the reason why d-LFcin17–30 had a stronger effect on the intramacrophagic growth of *M. avium* than LFcin17–30, and considering that peptide degradation is one of the factors that can have an impact on efficacy, we evaluated by high-performance liquid chromatography (HPLC) the kinetics of degradation of both peptides in the presence of the cell culture medium used in the infection assays. As expected, the peptide composed of amino acids in the d-form was significantly more resistant to degradation, persisting with no more than 30% degradation for up to 96 h of incubation, whereas 50% of the l-form of the peptide was degraded after 24 h of incubation, being completely degraded after 96 h (Fig. 1C).

### Lactoferricin peptides do not colocalize with *M. avium* inside macrophages.

**FIG 2:**
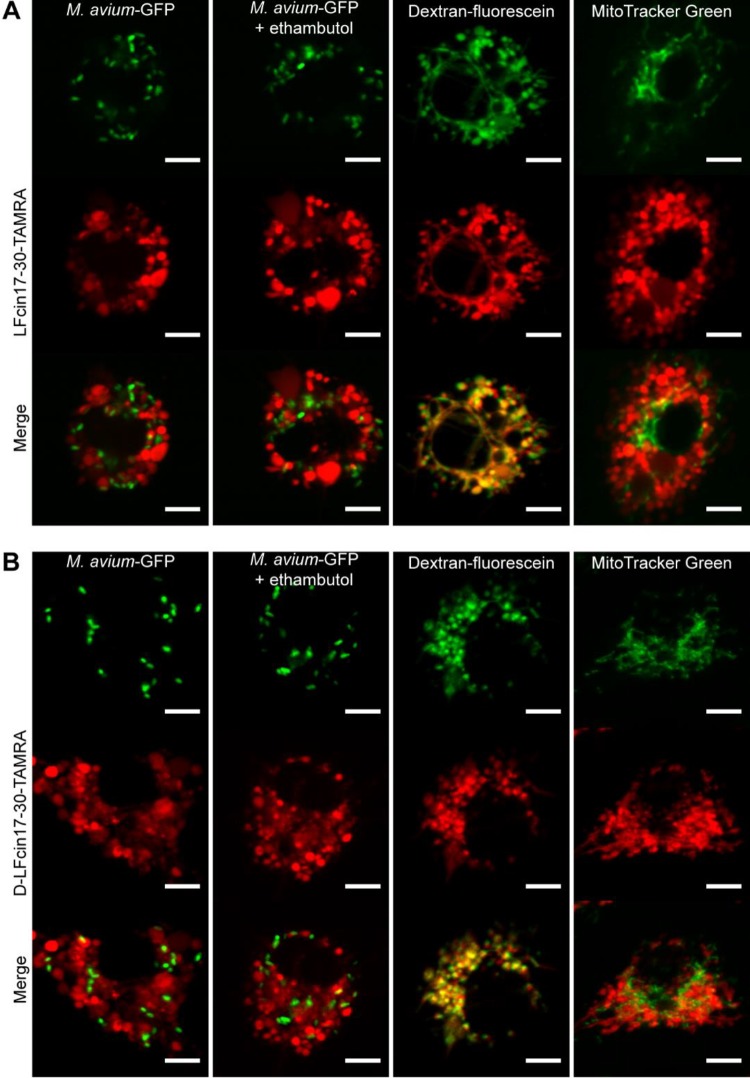
Intracellular distribution and localization of lactoferricin peptides in *M. avium*-infected macrophages. The figure shows live-cell imaging of BALB/c BMM infected with *M. avium* and treated with 10 µM red fluorescent peptide for 2 h: LFcin17–30—TAMRA (A) or d-LFcin17–30—TAMRA (B). First column, *M. avium*-GFP-infected macrophages; second column, *M. avium*-GFP-infected macrophages treated with 7.2 µM ethambutol for 2 h; third column, *M. avium* 2447 SmT-infected macrophages incubated with 22.5 µM fluorescein-conjugated dextran for 2 h; forth column, *M. avium* 2447 SmT-infected macrophages incubated with 200 nM MitoTracker Green for 30 min. One representative cell of one representative experiment out of three is shown for each condition. Scale bar, 5 µm.

In order to understand the mechanisms by which lactoferricin peptides inhibited the intramacrophagic growth of *M. avium*, we characterized the intracellular distribution of the peptides inside *M. avium*-infected macrophages. For that, we used peptides labeled with TAMRA [5(6)-carboxytetramethylrhodamine, a rhodamine derivative], a strain of *M. avium* expressing green fluorescent protein (GFP), and fluorescein-labeled markers of endosomes or mitochondria. Figure 2 depicts representative pictures of macrophages 2 h after infection with *M. avium* and peptide treatment. LFcin17–30 (Fig. 2A) and d-LFcin17–30 (Fig. 2B) exhibited similar distributions inside macrophages, and neither colocalized with *M. avium*. The exclusion of the peptides from mycobacterium-containing vesicles was not altered by the treatment with ethambutol (Fig. 2A and B, second column), by the incubation time (20 min for up to 24 h [data not shown]), or the time of peptide addition, either immediately after infection (Fig. 2) or 4 to 5 days after infection (data not shown). Because the intracellular distribution of both peptides had a vesicular appearance, we studied their colocalization with the endocytic pathway. For that, *M. avium*-infected macrophages were coincubated with peptides and dextran-fluorescein isothiocyanate (FITC) for 2 h, and we found that both peptides extensively colocalized with endosomes (Fig. 2A and B, third column), suggesting that they are internalized by this pathway. Importantly, neither LFcin17–30 nor d-LFcin17–30 significantly localized with mitochondria, which indicates that they will not exert a toxic effect on this organelle (Fig. 2A and B, fourth column). The evaluation of macrophage viability by resazurin reduction also indicated that the TAMRA-labeled peptides had no toxicity toward the macrophages under the conditions of the assay (data not shown).

### Lactoferricin peptides increase macrophage production of proinflammatory cytokines.

**FIG 3:**
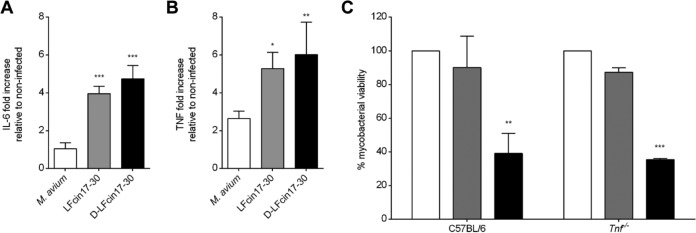
Roles of cytokines in the antimycobacterial activities of lactoferricin peptides. Twenty-four hours after infection and treatment with 40 µM LFcin17–30 (gray) or d‑LFcin17–30 (black), the levels of IL-6 (A) and TNF-α (B) were determined in the supernatant of BALB/c BMM. The graphs represent the averages + standard deviations of results from three independent experiments, presented as the fold increase relative to their levels in noninfected control macrophages. Statistical analysis was performed using one-way ANOVA with Tukey’s multiple-comparison test. *, *P* < 0.05; **, *P* < 0.01; ***, *P* < 0.001 compared to nontreated wells. (C) *M. avium* 2447 SmT growing inside C57BL/6 and Tnf^−/−^ BMM were treated with 40 µM LFcin17–30 (gray) or d-LFcin17–30 (black). After 5 days of incubation, bacteria were quantified by a CFU assay. The graph represents the averages from two independent experiments, expressed as the percentage of growth of mycobacteria in each well relative to the growth of mycobacteria in the nontreated infected wells (control) in each experiment. Statistics were performed using two-way ANOVA with Tukey’s multiple-comparison test. **, *P* < 0.01; ***, *P* < 0.001 compared to nontreated wells (control).

Considering that lactoferricin peptides appeared to decrease *M. avium* viability inside macrophages without a direct interaction with the bacteria (Fig. 2), we questioned whether they had a modulatory effect on macrophage function. For that, we used macrophage supernatants to measure the levels of several cytokines 24 h after infection with *M. avium* and concomitant treatment with the peptides. The treatment with lactoferricin peptides significantly increased the production of interleukin 6 (IL-6) (Fig. 3A) and TNF-α (Fig. 3B) by BMM infected with *M. avium* (but not by noninfected macrophages [data not shown]), with no significant differences between the two peptides. IL-1β, IL-10, CCL2, IL-12p40, and IFN-γ were not significantly induced either by *M. avium* infection or by peptide treatments (data not shown).

### **The antimicrobial effects of lactoferricin peptides inside macrophages are not dependent on the production of TNF-**α** and/or of IL-6 by macrophages.**

Both peptides increased the production of TNF-α by *M. avium*-infected macrophages, and macrophage activation by TNF-α can lead to intracellular killing of mycobacteria (19, 20); therefore, we tested whether this cytokine was necessary for the antibacterial effect of the peptides. We took BMM from Tnf^−/−^ mice and from congenic C57BL/6 wild-type mice, infected them with *M. avium* 2447 SmT, treated them with LFcin17–30 or d-LFcin17–30, and measured *M. avium* growth after 5 days. Our results, presented in Fig. 3C, showed that, similarly to what was observed before in BALB/c macrophages (Fig. 1D), only d-LFcin17–30 significantly inhibited the growth of *M. avium* inside its host cell (Fig. 3C). Strikingly, the effects of d‑LFcin17–30 on *M. avium* intracellular growth were similar for C57BL/6 and Tnf^−/−^ BMM, leading us to conclude that TNF-α is not necessary for the antibacterial effect of this peptide. By measuring cytokine levels in macrophage supernatants, we confirmed not only that Tnf^−/−^ BMM did not produce TNF-α but also that these macrophages did not produce significant amounts of IL-6, showing that the effect of the peptide is also IL-6 independent (data not shown).

### d-LFcin17–30 induces ultrastructural alterations on *M. avium*-infected macrophages.

**FIG 4:**
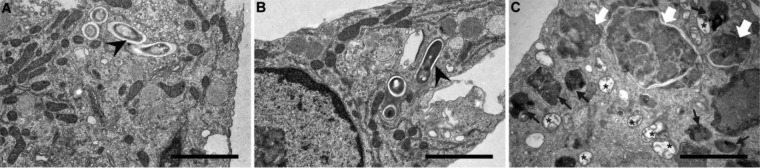
Ultrastructural alterations induced by lactoferricin peptides on *M. avium*-infected macrophages. Transmission electron microscopy of BALB/c BMM infected with *M. avium* 2447 SmT (A) and treated with 40 µM LFcin17–30 (B) or d-LFcin17–30 (C) for 5 days. Scale bar, 2 µm. Symbols: black arrowheads, intact mycobacteria; black arrows, dense and multivesicular bodies; white arrow, large dense structures probably resulting from multivesicular fusion and digestion; asterisk, double-membrane vesicles.

To gain an in-depth knowledge of the mechanisms by which d-LFcin17–30 inhibits mycobacterial growth, transmission electron microscopy (TEM) was performed on *M. avium*-infected macrophages treated with the lactoferricin peptides. Representative images of these assays are shown in Fig. 4. Striking alterations in macrophage ultrastructure were evident when they were treated with d-LFcin17–30 (Fig. 4C). As was expected, intact mycobacteria were difficult to detect, whereas in nontreated macrophages or even in LFcin17–30-treated macrophages, intact mycobacteria were visualized (Fig 4A and B, arrowheads). Several double-membrane vesicles containing digested material, suggestive of autophagosomes (Fig. 4C, asterisks), were observed in d-LFcin17–30-treated macrophages. A high number of dense vesicles and multivesicular bodies loaded with dense material were also seen (Fig. 4C, black arrows). Large structures, exhibiting several membranes and delimitations inside, suggestive of cell material ingestion and fusion with endosomes, lysosomes, or autophagosomes (Fig. 4C, white arrows), were frequently seen. These alterations were not observed in the case of cells treated with LFcin17–30 (Fig. 4B). These observations suggested that d-LFcin17–30 induced significant alterations in the macrophage vesicular traffic and membrane digestion pathways, which might contribute to mycobacterial killing.

### *M. avium*-infected macrophages have increased lysosomal content and autophagic vesicles upon d-LFcin17–30 treatment.

**FIG 5:**
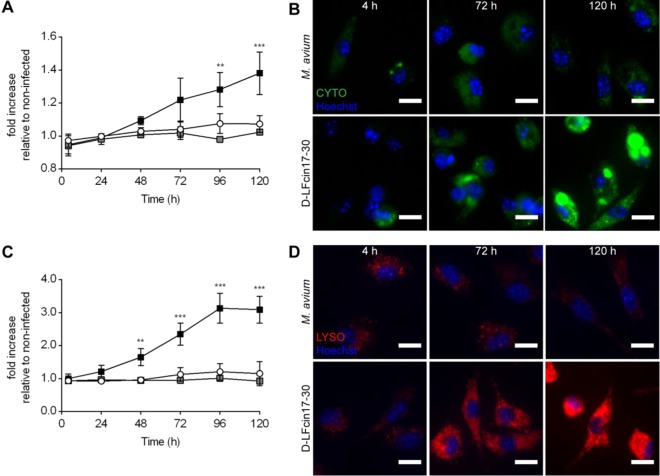
Autophagic and lysosomal content of *M. avium*-infected macrophages treated with lactoferricin peptides. At the end of 4, 24, 48, 72, 96, and 120 h of infection with *M. avium* 2447 SmT and treatment with d-LFcin17–30, macrophages were incubated with CYTO-ID (A, B) or LYSO-ID (C, D) detection kits for 30 min at 37°C. (A, C) The results represent the averages from three independent experiments, expressed as the fold increase in the fluorescence level of each detection reagent under each condition and time point relative to the fluorescence level of the noninfected nontreated well at 4 h. Statistics were performed using two-way ANOVA with Tukey’s multiple-comparison test. **, *P* < 0.01; ***, *P* < 0.001 compared to nontreated infected wells. White circles, *M. avium*-infected macrophages; gray squares, *M. avium*-infected and LFcin17–30-treated macrophages; black squares, *M. avium*-infected and d-LFcin17–30-treated macrophages. (B, D) Representative pictures of one experiment out of three of *M. avium*-infected macrophages (top) and treated with d-LFcin17–30 (bottom) at 4, 72, and 120 h with CYTO-ID (B) or LYSO-ID (D). Scale bar, 10 µm.

Given the striking morphological alterations induced by d-LFcin17–30 on *M. avium*-infected macrophages (Fig. 4C) and the known role of cellular processes such as apoptosis, autophagy, and lysosomal fusion in the macrophage-mycobacterium interaction (13, 43), we sought to quantitatively evaluate these processes in live-cell experiments. Macrophages were infected with *M. avium* 2447 SmT and treated with either LFcin17–30 or d-LFcin17–30. After 4, 24, 48, 72, 96, and 120 h of incubation, the cells were analyzed for the three above-mentioned parameters (Fig. 5). We observed no significant changes in the levels of apoptosis or necrosis under any of the tested conditions, including treatment with either LFcin17–30 or d-LFcin17–30 (data not shown). In order to evaluate the levels of autophagy, we used the CYTO-ID kit, which is based on a cationic amphiphilic tracer dye that labels vacuoles associated with the autophagy pathway and should not accumulate within lysosomes (44). When we measured the total fluorescence intensity associated with autophagic vesicles, we saw that d-LFcin17–30 slightly but significantly increased the macrophages’ autophagic-vesicle content (Fig. 5A and B). Regarding the evaluation of the lysosomal content, macrophages treated with d-LFcin17–30 had a 3-fold increase in density levels of the LYSO dye, which accumulates in live acidic organelles, such as lysosomes (Fig. 5C and D). d-LFcin17–30 induced a similar increase in the lysosomal content of noninfected macrophages (data not shown), indicating that this effect is independent of mycobacterial infection. In agreement with the TEM results (Fig. 4), large vesicles could be seen in cells treated with d-LFcin17–30, and these were labeled with both LYSO and CYTO probes (Fig. 5B and D), indicating that these structures can exhibit both lysosome and autophagosome features.

## DISCUSSION

In this work, we show that lactoferricin-based antimicrobial peptides strongly inhibit the growth of *M. avium* inside its natural host cell, the macrophage, enhancing the effect of the conventional antibiotic ethambutol. Moreover, we show that the d-enantiomer of lactoferricin, d-LFcin17–30, activates lysosomal and autophagic pathways in the macrophage, which can be crucial for its capacity to kill intracellular mycobacteria.

In a previous work, we showed that LFcin17–30, its variants with arginines replaced with lysines and vice versa (LFcin17–30 all K and LFcin17–30 all R), and its d-enantiomer (d-LFcin17–30) were all active against *M. avium* in axenic cultures (40). In contrast, we now verify that only d-LFcin17–30 induces a significant decrease in mycobacterial growth inside macrophages. However, all peptides were effective when combined with the antibiotic ethambutol. The combination of antimicrobial peptides with conventional antibiotics is of great potential interest, as it might reduce the dosages of each compound, diminish the probability of resistance, and reduce the treatment time. In the clinics, ethambutol is used in combination with other antimycobacterial drugs, not only as a strategy to prevent the appearance of resistant strains but also due to its high toxicity when given alone in high doses (9, 41). The advantageous combination of ethambutol and iron chelators in the control of *M. avium* growth inside macrophages was recently reported (45). Ethambutol acts by impairing the biosynthesis of the cell wall, increasing cell permeability, and potentiating the actions of other drugs (9, 46, 47). The improvement in the antimycobacterial activity observed in the present work when ethambutol was administered together with the peptides is probably related to increased membrane permeability induced by either ethambutol, the peptides, or both, allowing for higher concentrations of the compounds to enter the cell and potentiating their mutual activities.

Given that d-LFcin17–30 was more active than the l-peptides, we proceeded to investigate the mechanism by which the d-enantiomer of LFcin17–30 exerts its antimycobacterial activity inside macrophages. Due to their peptidic nature, AMP are highly susceptible to proteases and other plasma components. This feature is one of the obstacles to AMP application in the clinics, as it results in low stability and bioavailability, limiting most current AMP applications to topical agents (48). One strategy employed to overcome this problem is the use of nonnatural d-enantiomers of amino acids, as they are more resistant to proteolytic activity (49). In fact, several reports describe that AMP, including lactoferricin derivatives, composed of d-amino acids are more resistant to degradation and have activities higher than or similar to those of their counterparts with l-amino acids (50–56). In the case of the peptides studied in this work, d‑LFcin17–30 was capable of resisting degradation, and it persisted in cell culture medium at a higher concentration than LFcin17–30 over time, indicating that this is probably a crucial factor for its higher antimycobacterial activity. The fact that the d-enantiomer is more active than the l-enantiomer also reveals that the observed antimicrobial effect is probably not related to chiral receptors, because they would not recognize d-amino acids.

Because we had reported previously that these peptides exhibit a direct antimicrobial effect against *M. avium* in broth culture (40), we initially hypothesized that the inhibition of mycobacterial growth inside macrophages is the result of a direct effect on the mycobacteria. However, when we studied the distribution and subcellular localization of LFcin17–30 and d-LFcin17–30 inside *M. avium*-infected macrophages, we saw no colocalization between AMP and bacteria. We performed all the assays in live cells with fluorochrome-labeled peptides to avoid fixation-related artifacts, but we failed to detect any colocalization, even in the presence of ethambutol, at any incubation time. The peptides seemed to follow an endocytic pathway, colocalizing with fluorescein-conjugated dextran. Although we cannot exclude a possible alteration in peptide distribution caused by the fluorochrome link, the additional assays performed clearly indicate that d-LFcin17–30 impacts macrophage biology, and this may cause mycobacterial killing rather than having a direct action on the bacteria.

The administration of LFcin17–30 and d-LFcin17–30 was accompanied by increased levels of TNF-α and IL-6 production by *M. avium*-infected macrophages. TNF-α is necessary for the hosts' resistance to *M. avium*. This cytokine is involved in macrophage activation, being able to induce intracellular killing of mycobacteria (19, 20, 57, 58). In turn, IL-6 is a cytokine involved in the modulation of inflammation and the acute-phase response, important for host responses to mycobacterial infections (59). Although both peptides increased the levels of TNF-α and IL-6, these are not essential for the antimicrobial effect of d-LFcin17–30, as their absence did not interfere with the peptide’s effect. We did not detect increased production of nitrite in d-LFcin17–30-treated macrophages (data not shown). Furthermore, we did not expect nitric oxide to be involved in the antimycobacterial effect of d-LFcin17–30, since we have previously shown that oxygen- and nitrogen-reactive species are not important for the control of *M. avium* growth inside murine macrophages (24, 25).

Lactoferricin has been reported to have multiple roles in the host immune response. Besides having a direct antimicrobial activity on several pathogens, lactoferricin can inhibit septic shock by binding to endotoxins (60). Additionally, it has been shown to selectively kill cancer cells (61–66) in a process involving both apoptosis and autophagy (66). Autophagy is a host cell effector mechanism used as a quality control for the removal of protein aggregates and damaged organelles. Under stress conditions, the cell can activate autophagy for survival, selectively targeting different cargos for degradation. Xenophagy, the autophagic degradation of intracellular pathogens, is an innate defense weapon used by a host to control pathogen replication and proliferation (67). In the case of mycobacterial infections, vitamin D_3_ concomitantly induces the production of antimicrobial peptides (such as cathelicidin) and autophagy, both of which play a role in the control of the pathogen’s growth within macrophages (32–34). Interestingly, the peptide Beclin-1 was shown to control mycobacterial growth inside macrophages by inducing autophagy (68), and the d-form of the peptide induces higher activation of this pathway (69).

The ultrastructural changes observed in this work when *M. avium*-infected macrophages were treated with d-LFcin17–30, together with the increase in lysosomal and autophagic vesicles, lead us to conclude that the peptide facilitates the targeting of mycobacteria to lysosomal degradation. Given that d-LFcin17–30 is composed of nonnatural d-amino acids, the cells may recognize the peptide as a stress signal, leading to downstream activation of inflammatory pathways. We cannot clearly distinguish whether autophagy or phagosomal maturation is being activated. These two pathways overlap and can have common denominators (e.g., human VPS34 and RAB7) (8, 67, 70). Either way, we postulate that lactoferricin primes mycobacteria for vesicular digestion, having phagosomes or autophagosomes fusing with lysosomes for cargo degradation.

In summary, in this work, we showed that a d-enantiomer of lactoferricin, d-LFcin17–30, modulates macrophage activity toward a state which favors mycobacterial elimination. This observation, together with the data on the safe use of lactoferricin peptides to improve animal health in different mouse models (64, 71, 72), opens the way toward a possible use of this peptide to treat mycobacterial infections as an adjunct therapy with conventional antibiotics. Additionally, these data suggest other possible applications for d-LFcin17–30 in situations requiring macrophage activation.

## MATERIALS AND METHODS

### Peptides.

Bovine lactoferricin peptides (LFcin17–30, d-LFcin17–30, LFcin17–30 all K, and LFcin17–30 all R) (Table 1) were synthesized by solid-phase peptide synthesis using 9-fluorenyl-methoxycarbonyl (Fmoc) chemistry with a Syro II synthesizer (Biotage, Uppsala, Sweden) as described previously (73). Peptide synthesis-grade solvents were obtained from Actu-All Chemicals (Oss, The Netherlands), the preloaded NovaSyn TGA resins from Novabiochem (Merck Schuchardt, Hohenbrunn, Germany), and the *N*-α-Fmoc-amino acids from ORPEGEN Pharma (Heidelberg, Germany) and Iris Biotech (Marktredwitz, Germany). LFcin17–30 and d-LFcin17–30 were labeled in synthesis with 5(6)-carboxytetramethylrhodamine (TAMRA; Novabiochem) by coupling TAMRA to the ε-amino group of an additional C-terminal lysine residue using Fmoc-Lys(ivDde)-OH, resulting in a labeling stoichiometry of 1:1, without any free TAMRA remaining. Briefly, the peptide was synthesized as described above on *N*-α-Fmoc-*N*-ε-1-(4,4-dimethyl-2,6-dioxocyclohex-1-ylidene)-3-methylbutyl-l-lysine coupled to NovaSyn TGR resin (Novabiochem) with the N-terminal amino acid protected by *N*-α-*tert*-butoxycarbonyl. Subsequently, the ivDde-protecting group at the C-terminal Lys was released by hydrazinolysis (2% hydrazine hydrate in *N*-methyl-2-pyrrolidone [NMP]) followed by overnight incubation with 1.5 eq TAMRA in (NMP) containing 1.5 eq of 1-hydroxybenzotriazole (HOBt), 1.7 eq of 2-1[H-benzotriazole-1-yl]-1,1,3,3-tetramethylaminium tetrafluoroborate (TBTU), and 70 μl of *N*,*N*-diisopropylethylamine (DIPEA) in a final volume of 2 ml. Next, the peptide-containing resin was washed twice with NMP and twice with 20% piperidine, followed by three consecutive washes with NMP, isopropyl alcohol (IPA), and dichloromethane (DCM). Subsequently, the peptide was detached from the resin and deprotected as described previously (73).

Peptides were purified to a purity of at least 95% by semipreparative reverse-phase HPLC (RP-HPLC) (JASCO Corporation, Tokyo, Japan) on a Vydac C_18 _column (catalog number 218MS510; Vydac, Hesperia, CA, USA), and the authenticity of the peptides was confirmed by matrix-assisted laser detection ionization–time of flight (MALDI-TOF) mass spectrometry on a Microflex LRF mass spectrometer equipped with an additional gridless reflectron (Bruker Daltonik, Bremen, Germany) as described previously (73).

All purified peptides were freeze-dried. Peptide stock solutions were prepared in phosphate-buffered saline (PBS; pH = 7.4), with 10% dimethyl sulfoxide (DMSO) in the case of the labeled peptides, and stored at −20°C until use.

### HPLC.

Peptides (LFcin17–30 and d-LFcin17–30) were incubated with Dulbecco’s modified Eagle’s medium (DMEM), supplemented as stated below, at 37°C for 4 days. At the end of 0, 0.5, 2, 4, 8, 24, 48, 72, and 96 h, an aliquot was taken from each mixture and analyzed by high-performance liquid chromatography (HPLC). The HPLC (Hitachi Elite Autosampler L-2200, pump L-2130, diode array detector L-2455, and column oven L-2300) was performed with a 150-mm-diameter C_18_ reverse-phase column (Merck). Each analysis involved an injection volume of 40 µl and elution with 0 to 100% acetonitrile in 0.05% aqueous trifluoroacetic acid (TFA) at a flow rate of 1 ml/min; the detection wavelength was set to 220 nm. The chromatograms were analyzed with EZChrom Elite software, and the peaks were integrated to extract the area.

### Bacteria.

In this work, two strains of *Mycobacterium avium* were used: (i) *M. avium* strain 2447 smooth transparent variant (SmT), originally isolated by F. Portaels (Institute of Tropical Medicine, Antwerp, Belgium) from an AIDS patient, and (ii) *M. avium* 104:pMV306 (*hsp60 gfp*) expressing green fluorescent protein (*M. avium-*GFP) (74). Mycobacteria were grown and stored as described previously (40).

### BMM.

Macrophages were derived from the bone marrow of male BALB/c, C57BL/6, and C57BL/6 TNF-α-deficient (Tnf^*−/−*^) mice bred at the i3S/IBMC animal facility. TNF-α-deficient breeder mice were originally purchased from B & K Universal (East Yorkshire, UK). Bone marrow-derived macrophages (BMM) were obtained as described previously (75).

### Macrophage infection and quantification of bacterial growth.

BMM at day 10 of culture were infected with 10^6^ CFU of *M. avium* 2447 SmT for 4 h at 37°C in a 7% CO_2_ atmosphere. After incubation, cells were washed several times to remove noninternalized bacteria and reincubated with new medium with or without 40 µM peptide (76.9 µg/ml LFcin17–30 and d-LFcin17–30, 73.6 µg/ml LFcin17–30 all R, 80.3 µg/ml LFcin17–30 all R), alone or in combination with the antibiotic ethambutol (2 µg/ml or 7.2 µM ethambutol dihydrochloride; Sigma-Aldrich, St. Louis, MO, USA). Each condition was tested in triplicate. After 5 days in culture, the intracellular growth of *M. avium* 2447 SmT was evaluated by determining the number of CFU, as described previously (75).

### Measurement of macrophage viability.

The viability of BALB/c BMM was determined by resazurin reduction. After 24 h of infection and peptide treatment, the supernatant was removed and macrophages were incubated with new medium containing 125 µM resazurin (Sigma-Aldrich, St. Louis, MO, USA) for 24 h at 37°C in a 7% CO_2_ atmosphere. The fluorescence of resorufin, resulting from the conversion from resazurin by metabolically active cells, was measured at an excitation wavelength (λ_ex_) of 560 nm and an emission wavelength (λ_em_) of 590 nm.

### Peptide’s distribution and localization inside macrophages.

BALB/c BMM were cultured in μ-Slide 8-well plates (ibidi GmbH, Germany). At the 10th day of culture, macrophages were infected with either *M. avium*-GFP or *M. avium* 2447 SmT and treated with 10 µM LFcin17–30—TAMRA or d‑LFcin17–30—TAMRA. Simultaneously, half of the *M. avium*-GFP-infected macrophages were treated with 7.2 µM ethambutol. Fluorescein-conjugated dextran (molecular weight, 10,000) (22.5 µM, final concentration) (Molecular Probes, Invitrogen, Carlsbad, CA, USA) or MitoTracker Green FM (200 nM, final concentration) (Molecular Probes, Invitrogen, Carlsbad, CA, USA) was added to *M. avium* 2447 SmT-infected macrophages for endosomal or mitochondrial labeling, respectively. Fluorescein-conjugated dextran was added along with the peptides immediately after infection and incubated for 2 h, whereas MitoTracker Green FM was incubated for 30 min prior to visualization. Macrophages were observed and photographed live, using a Leica TCS SP5II laser scanning confocal microscope (Laser Microsystems, Germany) with a 63× oil objective. Immediately before visualization, cells were washed with PBS and kept in RPMI medium without phenol red (Life Technologies, Inc., Paisley, UK).

### Cytokine production.

Cytokine production was evaluated in the supernatants of macrophage cultures 24 h after infection with *M. avium* 2447 SmT and peptide treatment. The levels of six different cytokines (IL-12p70, TNF-α, IFN-γ, CCL2, IL-10, and IL-6) were determined using the BD cytometric bead array (CBA) mouse inflammation kit (BD Biosciences, San Jose, CA, USA) according to the manufacturer’s instructions. Briefly, standards and samples were incubated for 2 h with a mixture of capture beads for each cytokine and with a mixture of phycoerythrin (PE)-conjugated antibodies as a detection reagent. Afterward, the wells were washed, the supernatant was discarded, and the beads were resuspended in wash buffer. The standards and samples were then acquired in a BD FACSCanto II cytometer (BD Biosciences, San Jose, CA, USA) and the results analyzed using the FCAP Array software (BD Biosciences, San Jose, CA, USA).

### Transmission electron microscopy.

In brief, BALB/c BMM infected with *M. avium* 2447 SmT and treated with lactoferricin peptides for 5 days were fixed with 2.5% glutaraldehyde (Electron Microscopy Sciences, Hatfield, PA, USA) and 2% paraformaldehyde (Merck, Darmstadt, Germany) in cacodylate buffer (0.1 M, pH 7.4) for 2 h at room temperature. Samples were dehydrated and embedded in Epon resin (TAAB, Berks, England). Ultrathin sections (40- to 60-nm thickness) were prepared on an RMC Ultramicrotome (Powertome, USA) using diamond knives (DDK, Wilmington, DE, USA). The sections were mounted on 200-mesh copper or nickel grids, stained with uranyl acetate and lead citrate for 5 min each, and examined under a JEOL JEM 1400 TEM (Tokyo, Japan). Images were digitally recorded using an Orius charge-coupled-device (CCD) digital camera (1,100 W; Gatan, Tokyo, Japan) at the HEMS/i3S of Universidade do Porto, Porto, Portugal.

### Live-cell imaging.

For live-cell imaging, BALB/c BMM were cultured on μ-Plate 96-well ibiTreat (ibidi GmbH, Germany) as stated above. After *M. avium* infection and treatment with lactoferricin peptides (time zero), the cells were incubated at 37°C in a 7% CO_2_ atmosphere, and at the end of 4, 24, 48, 72, 96, and 120 h, the levels of apoptosis and necrosis, the lysosomal content, and the autophagic levels were assessed separately. For that, Enzo Cellestial fluorescent probes were used from the apoptosis/necrosis detection kit, LYSO-ID detection kit, and CYTO-ID autophagy detection kit (Enzo Life Sciences Inc., USA). According to the manufacturer’s instructions, at each time point the cells were washed and incubated with the respective detection reagents for 30 min at 37°C in a 7% CO_2_ atmosphere. For visualization and image acquisition, macrophages were incubated with PBS-5% fetal bovine serum (FBS). Images were collected in a controlled environment (37°C and CO_2_ atmosphere) with a Nikon 40×/0.95-numerical-aperture (NA) Plan Fluor objective in a high-throughput automated fluorescence wide-field microscope (IN cell analyzer 2000; GE Healthcare, Little Chalfont, UK). The 2.5-dimensional (2.5-D) acquisition and deconvolution mode was used to integrate the signal over a 1.5-µm Z-section, generating a pseudo 3-D projection. Each well was screened for 10,000 nuclei (up to 72 fields). Quantification of the fluorescence levels (expressed as the mean density value of the pixels) of each detection reagent (apoptosis, necrosis, LYSO-ID, and CYTO-ID detection reagents) was performed with Developer Toolbox 1.9.2 (GE Healthcare, Little Chalfont, UK). Briefly, nuclear and cytoplasm segmentation algorithms were used to identify and quantify the number of cells under all conditions. The fluorescence level of each individual cell, under each condition and kit, was measured, allowing us to calculate the mean fluorescence value for each well.

### Statistical analysis.

Statistical analyses were performed with GraphPad Prism 6 (GraphPad Software, Inc., La Jolla, CA, USA) using two-way analysis of variance (ANOVA) with Tukey’s multiple-comparison test. Differences with a *P* value under 0.05 were considered significant (*, *P* < 0.05; **, *P* < 0.01; ***, *P* < 0.001).
